# Crystal structure and Hirshfeld surface analysis of 3-(4-meth­oxy­phen­yl)-2-[(thio­phen-2-yl)carbon­yl]acrylo­nitrile

**DOI:** 10.1107/S205698902601042X

**Published:** 2026-10-13

**Authors:** Elnur Z. Huseynov, Ayten S. Safarova, Farid N. Naghiyev, Malahat M. Kurbanova, Mehmet Akkurt, Jamshid M. Ashurov, Hamouda Adam Hamouda, İbrahim G. Mamedov

**Affiliations:** a Faculty of Chemistry, Baku State University, Z. Khalilov str. 23, Az 1148, Baku, Azerbaijan; b Department of Physics, Faculty of Sciences, Erciyes University, 38039 Kayseri, Türkiye; c Institute of Bioorganic Chemistry, Academy of Sciences of Uzbekistan, M. Ulugbek Str. 83, Tashkent 100125, Uzbekistan; d Department of Chemistry, Faculty of Science, University of Kordofan, Sudan; Institute of Chemistry, Chinese Academy of Sciences

**Keywords:** crystal structure, Knoevenagel condensation reaction, C—H⋯N inter­action, Hirshfeld surface analysis

## Abstract

In the title com­pound, C_15_H_11_NO_2_S, the intra­molecular C—H⋯O hy­dro­gen-bond inter­action forms an *S*(6) ring motif. The dihedral angle between the planes of the thio­phene and meth­oxy­phenyl ringss 12.26 (15)°. In the crystal, mol­ecular pairs are joined by C—H⋯N contacts, forming *R*_2_^2^(16) motifs lying parallel to the (001) plane. They are connected by C—O⋯π and π–π inter­actions [centroid-to-centroid lengths = 3.9049 (18) and 3.9050 (18) Å], resulting in layers parallel to the (100) plane. These layers are linked by C—H⋯N inter­actions. Hirshfeld surface analysis indicates that H⋯H (35.3%), O⋯H/H⋯O (13.5%), C⋯C (12.5%), N⋯H/H⋯N (11.6%), C⋯H/H⋯C (11.6%) and S⋯H/H⋯S (9.0%) inter­actions make significant contributions.

## Abstract 2

In the title crystal, mol­ecular pairs are connected by C—H⋯N contacts, producing *R*_2_^2^(16) motifs parallel to the (001) plane. Mol­ecules are linked by C—O⋯π and π–π inter­actions, forming layers parallel to the (100) plane. These layers are connected by C—H⋯N inter­actions.

## 1. Chemical context

Knoevenagel condensation reactions play a significant role in carbon–carbon bond formation, providing a versatile strategy for the synthesis of α-functionalized chalcone analogues and other conjugated mol­ecular frameworks (Naghiyev *et al.*, 2023 ▸; Mamedov & Khalilov, 2025 ▸; Abdullayeva *et al.*, 2026 ▸). Depending on the substrates and reaction conditions, these transformations can proceed under relatively mild conditions and afford products in good yields, sometimes with high stereoselectivity (Khalilov *et al.*, 2011 ▸; Khalilov *et al.*,2023 ▸; Tüzün *et al.*, 2025 ▸). Knoevenagel adducts have also been used as inter­mediates in the synthesis of com­pounds investigated in medicinal chemistry and materials research (Naghiyev *et al.*, 2022 ▸; Naghiyev *et al.*, 2024 ▸; Karimli *et al.*, 2023 ▸). Among these chalcone analogues, α,β-unsaturated nitrile derivatives are of particular inter­est because their structural diversity and reactive functional groups allow further synthetic transformations (Akkurt *et al.*, 2018 ▸; Asgarova *et al.*, 2019 ▸; Shikhaliyev *et al.*, 2025 ▸). Compounds combining a thio­phene ring with an α,β-unsaturated carbonyl group and a nitrile group provide substrates for such transformations. In particular, thio­phene-2-carbonyl-substituted acrylo­nitriles have been used in the synthesis of heterocyclic com­pounds.
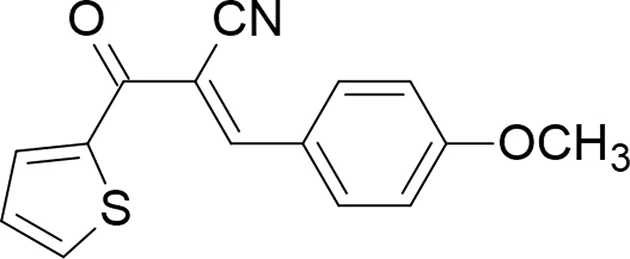


In the present study, 3-oxo-3-(thio­phen-2-yl)propane­nitrile was reacted with 4-meth­oxy­benzaldehyde in methanol at room tem­per­a­ture to afford the title com­pound (Fig. 1 ▸) and we now describe its structure. *N*-Methyl­piperazine served as a base, facilitating condensation at the activated methyl­ene group, followed by elimination of water. In this context, we report the crystal structure and Hirshfeld surface analysis of 3-(4-meth­oxy­phen­yl)-2-[(thio­phen-2-yl)carbon­yl]acrylo­nitrile as part of our ongoing investigations (Askerov *et al.*, 2020 ▸; Naghiyev *et al.*, 2021 ▸).

## 2. Structural commentary

The intra­molecular C8—H8⋯O1 hy­dro­gen-bond inter­action forms an *S*(6) ring motif in the mol­ecule (Table 1 ▸ and Fig. 2 ▸; Bernstein *et al.*, 1995 ▸). The dihedral angle between the planes of the thio­phene ring (S1/C1–C4) and the meth­oxy­phenyl ring (C9–C14) is 12.26 (15)°. The S1—C4—C5—C6, S1—C4—C5—O1, O1—C5—C6—C7, C7—C6—C8—C9 and C6—C8—C9—C14 torsion angles are 4.4 (5), 4.9 (4), −3.1 (5)° and −13.9 (5)°, respectively.

## 3. Supra­molecular features and Hirshfeld surface analysis

In the crystal, mol­ecular pairs are linked by C13—H13⋯N1 contacts, which form 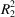
(16) motifs lying parallel to the (001) plane (Fig. 3 ▸). They are connected by the C—O⋯π contact [C5—O1⋯*Cg*1^*a*^: C5—O1 = 1.226 (3) Å, O1⋯*Cg*1^*a*^ = 3.721 (3) Å, C5⋯*Cg*1^*a*^ = 3.602 (3) Å and C5—O1⋯*Cg*1^*a*^ = 74.94 (17)°; symmetry code: (*a*) *x*, −*y* + 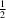, *z* − 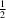; *Cg*1 is the centroid of the S1/C1–C4 thio­phene ring] and weak π–π inter­actions [*Cg*2⋯*Cg*2^*b*^ = 3.9049 (18) Å, slippage = 0.857 Å, *Cg*2⋯*Cg*2^*c*^ = 3.9050 (18) Å, slippage = 1.751 Å; symmetry codes: (*b*) *x*, −*y* + 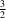, *z* − 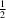; (*c*) *x*, −*y* + 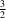, *z* + 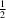; *Cg*2 is the centroid of the C9–C14 meth­oxy­phenyl ring], producing layers parallel to the (100) plane. These layers are linked by C13—H13⋯N1 inter­actions (Fig. 4 ▸).

In order to further characterize and qu­antify the inter­molecular inter­actions in the title com­pound, a Hirshfeld surface analysis was carried out using *CrystalExplorer21* (Spackman *et al.*, 2021 ▸). The Hirshfeld surface mapped over *d*_norm_ is illustrated in Fig. 5 ▸(*a*). The *d*_norm_ mappings ranged from −0.20 to +1.26 a.u. The shape index [Fig. 5 ▸(*b*)] shows paired red–blue regions typical of π–π stacking, with an additional weak signal in the area of the aromatic ring–O contact.

The fingerprint plots provide qu­anti­tative information about the noncovalent inter­actions in the crystal packing in terms of the percentage contribution of the inter­atomic contacts. The fingerprint plots (Fig. 6 ▸) show that H⋯H inter­actions (35.3%) contribute the most to surface contacts. Other notable inter­actions include O⋯H/H⋯O (13.5%), C⋯C (12.5%), N⋯H/H⋯N (11.6%), C⋯H/H⋯C (11.6%) and S⋯H/H⋯S (9.0%). Less significant inter­actions include S⋯O/O⋯S (1.2%) and O⋯N/N⋯O (0.4%).

## 4. Database survey

A search in the Cambridge Structural Database (CSD, Version 6.00, updated April 2025; Groom *et al.*, 2016 ▸) for the title com­pound reveals the five most closely related com­pounds, *viz*: (*E*)-2-(2-thien­yl)-3-(3,4,5-tri­meth­oxy­phen­yl)acrylo­nitrile [CSD refcode PEMBEP (I); Cobo *et al.*, 2006*a* ▸], (*E*)-3-(4-bromo­phen­yl)-2-(2-thien­yl)acrylo­nitrile [PEMBIT (II); Cobo *et al.*, 2006*a* ▸], (*E*)-3-phenyl-2-(3-thien­yl)acrylo­nitrile [PEMBOZ (III); Cobo *et al.*, 2006*a* ▸], (*Z*)-3-(4-methyl­phen­yl)-2-(3-thien­yl)acrylo­nitrile [QERPAF (IV); Cobo *et al.*, 2006*b* ▸] and (*Z*)-2-phenyl-3-(2,2′:5′,2′′-terthio­phen-3′-yl)acrylo­nitrile [UDUNAJ (V); Wagner *et al.*, 2007 ▸].

Compounds (I), (III), (IV) and (V) crystallize in the mononlinic space group *P*2_1_/*c*, whereas (II) crystallizes in the monoclinic space group *P*2_1_/*n*.

Compound (I) contains no direction-specific inter­molecular inter­actions. (II) exhibits orientational disorder of the thienyl fragment and the mol­ecules are linked into simple *C*(5) chains by a single C—H⋯N hy­dro­gen bond. In (III), the mol­ecules are linked into sheets by a combination of one C—H⋯N hy­dro­gen bond and one C—H⋯π(arene) hy­dro­gen bond. In (I)–(III), the nitrile com­ponents exhibit quite long C—C bonds [1.443 (2)–1.446 (4) Å] and very short C—N bonds [1.143 (4)–1.146 (4) Å]. The remaining bond distances in (I)–(III) show no unusual features. In (IV), the shortest nonbonded inter­molecular contacts between potential hy­dro­gen-bond donors and acceptors are all too long to be structurally significant. In crystal structure (V), the van der Waals forces seem to determine the packing. Weak C—H⋯N and C—H⋯π inter­actions also might be of some importance.

While the contributions of the strong inter­actions are quite consistent, weak inter­actions vary slightly depending on the mol­ecular conformation and the environment of the mol­ecules.

## 5. Synthesis and crystallization

A solution of 3-oxo-3-(thio­phen-2-yl)propane­nitrile (0.5 g, 3.31 mmol) and 4-meth­oxy­benzaldehyde (0.45 g, 3.31 mmol) in methanol (20 ml) was treated with a catalytic amount of *N*-methyl­piperazine (1–2 drops). The reaction mixture was stirred vigorously at room tem­per­a­ture for 3 h, and the course of the reaction was monitored by thin-layer chromatography (TLC; Silufol UV-254 plates; hexa­ne–ethyl acetate, 1:4 *v*/*v*). The resulting yellow crystals form directly; they were collected by filtration, washed with methanol and dried (m.p. 358 K; yield 91%).

^1^H NMR (300 MHz, DMSO-*d*_6_, ppm): 3.87 (*s*, 3H, CH_3_O), 7.14–8.15 (*m*, 7H, 4CH_arom_ + 3CH_thien­yl_), 8.29 (*s*, 1H, CH=). ^13^C NMR (75 MHz, DMSO-*d*_6_, ppm): 56.23 (CH_3_O), 105.74 (=C_quat_), 115.40 (2CH_arom_), 118.14 (CN), 124.85 (C_thien­yl_), 129.45 (CH_thien­yl_), 134.13 (2CH_arom_), 135.51 (CH_thien­yl_), 136.95 (CH_thien­yl_), 141.54 (C_arom_), 155.21 (CH=), 164.03 (O—C_arom_), 180.13 (C=O).

## 6. Refinement

Crystal data, data collection and structure refinement details are summarized in Table 2 ▸. All H atoms were placed in calculated positions and were refined as riding atoms with *U*_iso_(H) = 1.2*U*_eq_(C) or 1.5*U*_eq_(methyl C). The methyl H atoms were allowed to rotate, but not to tip, to best fit the experimental electron density.

## Supplementary Material

Crystal structure: contains datablock(s) I. DOI: 10.1107/S205698902601042X/nx2041sup1.cif

Structure factors: contains datablock(s) I. DOI: 10.1107/S205698902601042X/nx2041Isup2.hkl

Supporting information file. DOI: 10.1107/S205698902601042X/nx2041Isup3.cml

CCDC reference: 2594032

Additional supporting information:  crystallographic information; 3D view; checkCIF report

## Figures and Tables

**Figure 1:**
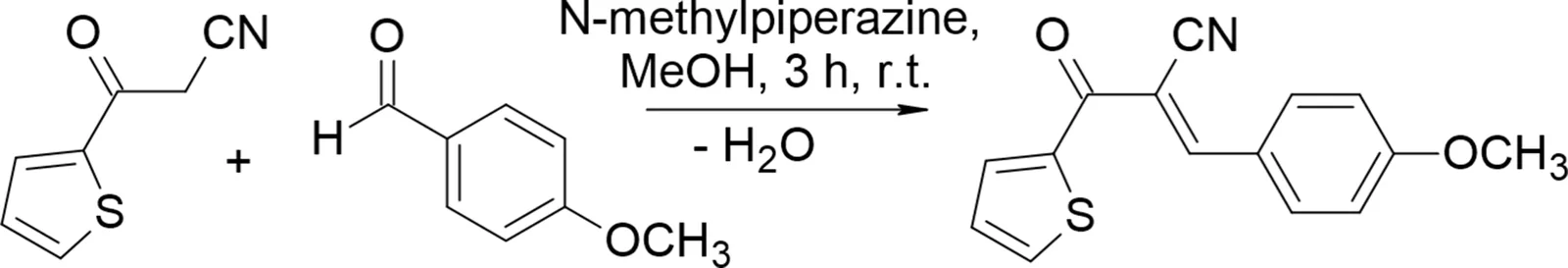
Synthesis of the title compound by Knoevenagel condensation.

**Figure 2:**
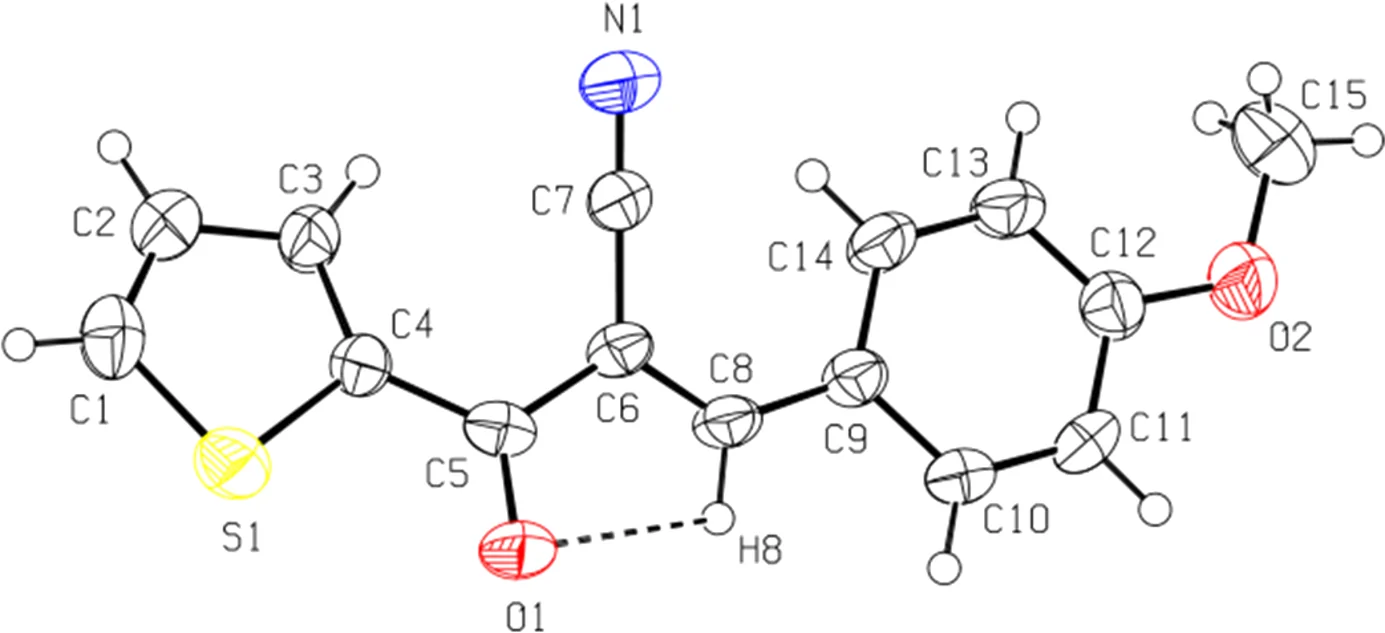
The mol­ecular structure showing the atomic labelling. Displacement ellipsoids are drawn at the 50% probability level. The intra­molecular C8—H8⋯O1 hydrogen bond is shown as a dashed line.

**Figure 3:**
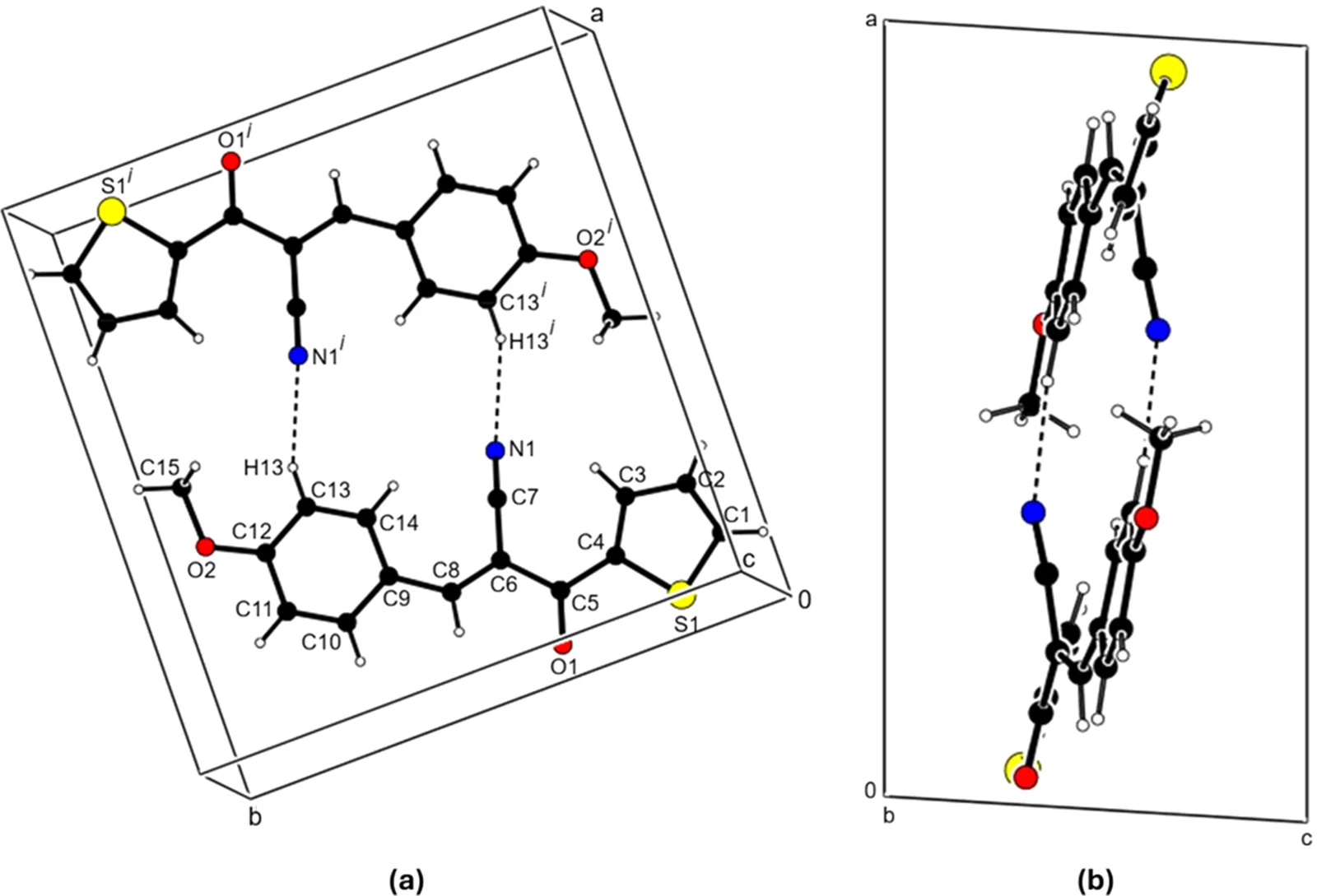
(*a*) A view of the C—H⋯N inter­actions in the unit cell. (*b*) A view down the *b* axis of the C—H⋯N inter­actions (dashed lines) for the situation shown in part (*a*). The 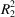
(16) graph-set motif formed by a pair of C—H⋯N inter­actions. [Symmetry code: (i) −*x* + 1, −*y* + 1, −*z* + 1.]

**Figure 4:**
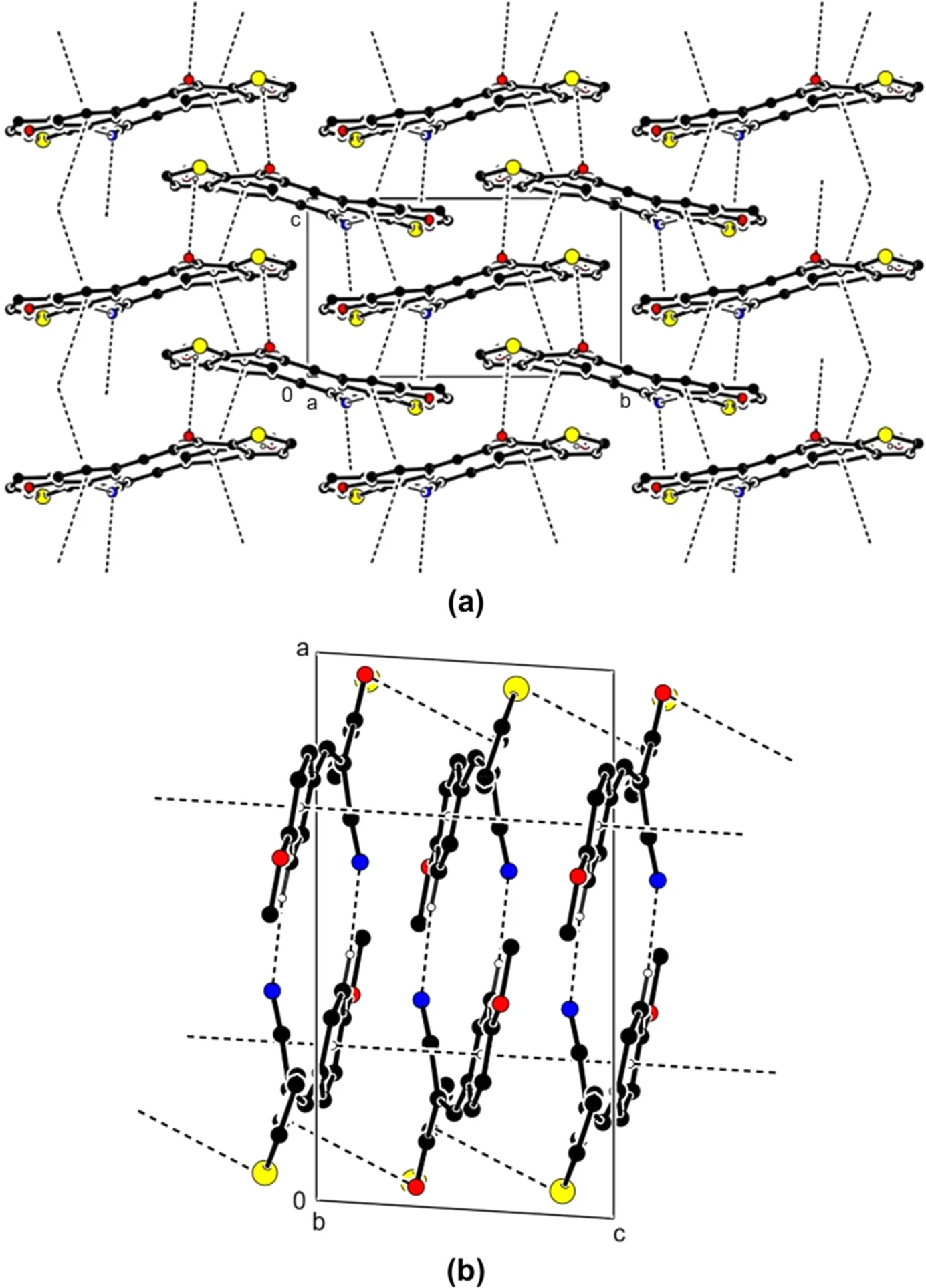
(*a*) A view of the C—O⋯π and π–π inter­actions (dashed lines) along the *a* axis. (*b*) A view of the C—H⋯N, C—O⋯π and π–π inter­actions (dashed lines) along the *b* axis. For clarity, H atoms not involved in hy­dro­gen bonding have been omitted.

**Figure 5:**
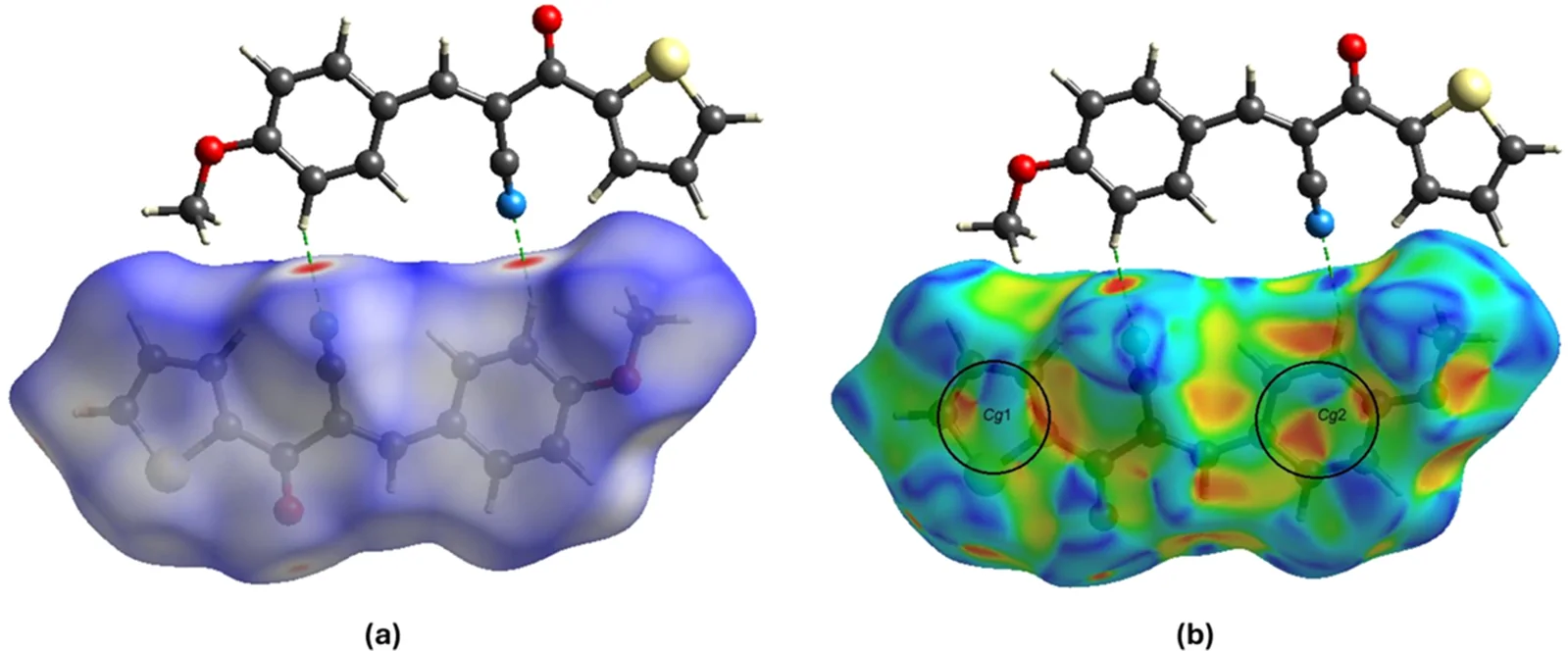
Hirshfeld surface of the title com­pound, mapped over (*a*) *d*_norm_ and (*b*) shape index, showing the inter­molecular C13—H13⋯N1 contacts.

**Figure 6:**
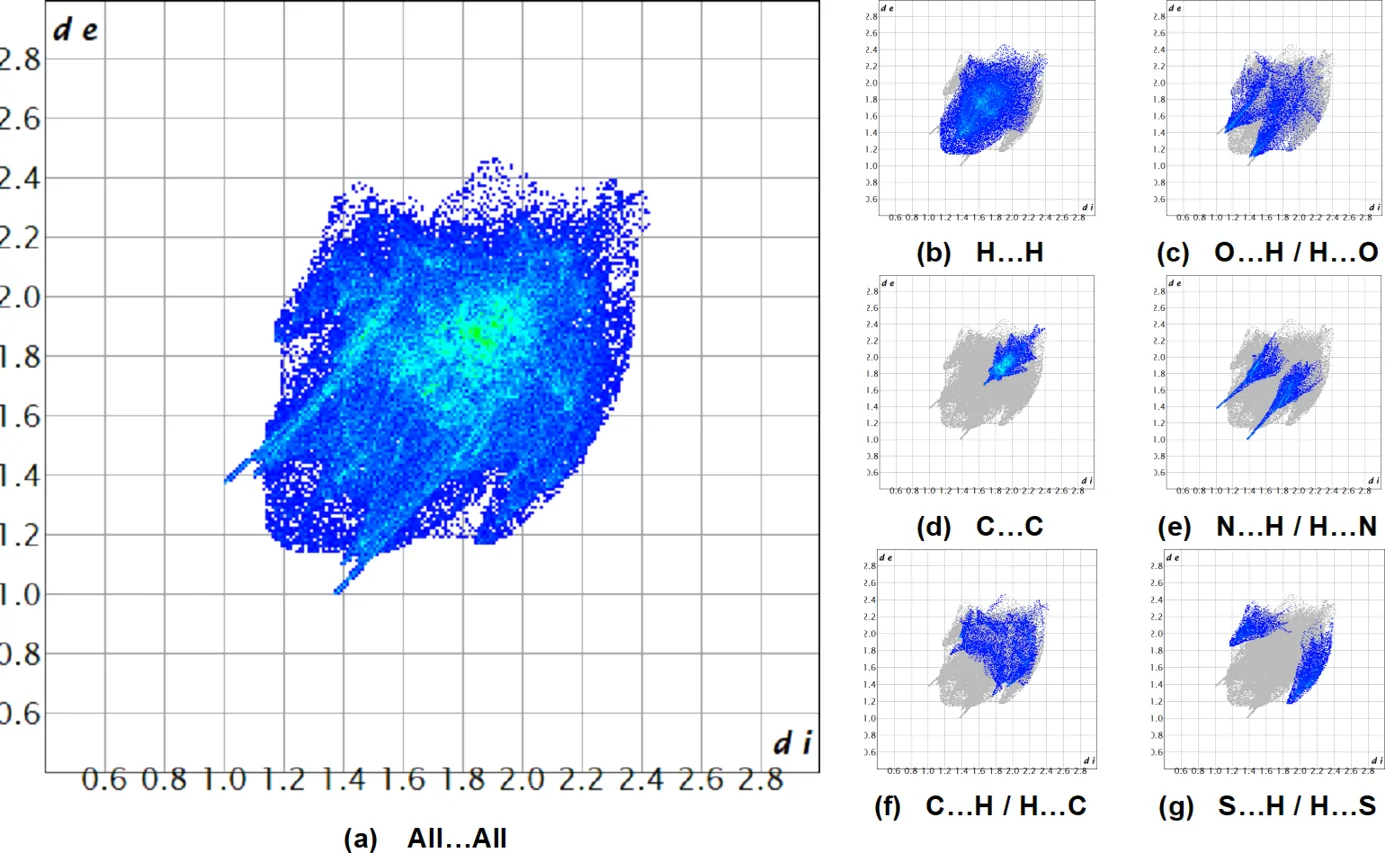
The two-dimensional fingerprint plots showing (*a*) all inter­actions, and those delineated into (*b*) H⋯H, (*c*) O⋯H/H⋯O, (*d*) C⋯C, (*e*) N⋯H/H⋯N, (*f*) C⋯H/H⋯C and (*f*) S⋯H/H⋯S inter­actions. The *d*_i_ and *d*_e_ values are the closest inter­nal and external distances (in Å) from given points on the Hirshfeld surface.

**Table 1:** Hydrogen-bond geometry (Å, °)

*D*—H⋯*A*	*D*—H	H⋯*A*	*D*⋯*A*	*D*—H⋯*A*
C8—H8⋯O1	0.93	2.40	2.779 (3)	104
C13—H13⋯N1^i^	0.93	2.51	3.394 (4)	158

Symmetry code: (i) 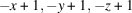.

**Table 2:** Experimental details

Crystal data	
Chemical formula	C_15_H_11_NO_2_S
*M*_r_	269.31
Crystal system, space group	Monoclinic, *P*2_1_/*c*
Temperature (K)	293
*a*, *b*, *c* (Å)	13.6250 (19), 13.0153 (18), 7.4161 (9)
β (°)	93.530 (4)
*V* (Å^3^)	1312.6 (3)
*Z*	4
Radiation type	Mo *K*α
μ (mm^−1^)	0.24
Crystal size (mm)	0.12 × 0.08 × 0.07

Data collection	
Diffractometer	Bruker APEXII CCD
Absorption correction	Multi-scan (*SADABS*; Bruker, 2012 ▸)
*T*_min_, *T*_max_	0.378, 0.745
No. of measured, independent and observed [*I* > 2σ(*I*)] reflections	21480, 2678, 1704
*R*_int_	0.095
(sin θ/λ)_max_ (Å^−1^)	0.625

Refinement	
*R*[*F*^2^ > 2σ(*F*^2^)], *wR*(*F*^2^), *S*	0.075, 0.139, 1.11
No. of reflections	2678
No. of parameters	173
H-atom treatment	H-atom parameters constrained
Δρ_max_, Δρ_min_ (e Å^−3^)	0.27, −0.30

Computer programs: *APEX2* (Bruker, 2012 ▸), *SAINT* (Bruker, 2012 ▸), *SHELXT* (Sheldrick, 2015*a* ▸), *SHELXL2014* (Sheldrick, 2015*b* ▸), *ORTEP-3 for Windows* (Farrugia, 2012 ▸) and *PLATON* (Spek, 2020 ▸).

## supplementary crystallographic information

### 3-(4-Methoxyphenyl)-2-[(thiophen-2-yl)carbonyl]prop-2-enenitrile Crystal data

**Table:**

C_15_H_11_NO_2_S	*F*(000) = 560
*M**_r_* = 269.31	*D*_x_ = 1.363 Mg m^−^^3^
Monoclinic, *P*2_1_/*c*	Mo *K*α radiation, λ = 0.71073 Å
*a* = 13.6250 (19) Å	Cell parameters from 4702 reflections
*b* = 13.0153 (18) Å	θ = 3.0–22.2°
*c* = 7.4161 (9) Å	µ = 0.24 mm^−^^1^
β = 93.530 (4)°	*T* = 293 K
*V* = 1312.6 (3) Å^3^	Block, yellow
*Z* = 4	0.12 × 0.08 × 0.07 mm

### 3-(4-Methoxyphenyl)-2-[(thiophen-2-yl)carbonyl]prop-2-enenitrile Data collection

**Table:**

Bruker APEXII CCD diffractometer	1704 reflections with *I* > 2σ(*I*)
φ and ω scans	*R*_int_ = 0.095
Absorption correction: multi-scan (SADABS; Bruker, 2012)	θ_max_ = 26.4°, θ_min_ = 3.0°
*T*_min_ = 0.378, *T*_max_ = 0.745	*h* = −16→17
21480 measured reflections	*k* = −16→16
2678 independent reflections	*l* = −8→9

### 3-(4-Methoxyphenyl)-2-[(thiophen-2-yl)carbonyl]prop-2-enenitrile Refinement

**Table:**

Refinement on *F*^2^	0 restraints
Least-squares matrix: full	Hydrogen site location: inferred from neighbouring sites
*R*[*F*^2^ > 2σ(*F*^2^)] = 0.075	H-atom parameters constrained
*wR*(*F*^2^) = 0.139	*w* = 1/[σ^2^(*F*_o_^2^) + (0.0373*P*)^2^ + 0.8766*P*] where *P* = (*F*_o_^2^ + 2*F*_c_^2^)/3
*S* = 1.11	(Δ/σ)_max_ < 0.001
2678 reflections	Δρ_max_ = 0.27 e Å^−^^3^
173 parameters	Δρ_min_ = −0.30 e Å^−^^3^

### 3-(4-Methoxyphenyl)-2-[(thiophen-2-yl)carbonyl]prop-2-enenitrile Special details

**Table:**

Geometry. All esds (except the esd in the dihedral angle between two l.s. planes) are estimated using the full covariance matrix. The cell esds are taken into account individually in the estimation of esds in distances, angles and torsion angles; correlations between esds in cell parameters are only used when they are defined by crystal symmetry. An approximate (isotropic) treatment of cell esds is used for estimating esds involving l.s. planes.

### 3-(4-Methoxyphenyl)-2-[(thiophen-2-yl)carbonyl]prop-2-enenitrile Fractional atomic coordinates and isotropic or equivalent isotropic displacement parameters (Å^2^)

**Table:**

	*x*	*y*	*z*	*U*_iso_*/*U*_eq_	
C1	0.1153 (3)	0.0523 (3)	0.3757 (4)	0.0547 (10)	
H1	0.092826	−0.015035	0.364876	0.066*	
C2	0.2084 (3)	0.0778 (3)	0.4318 (5)	0.0532 (9)	
H2	0.256854	0.029829	0.464225	0.064*	
C3	0.2242 (2)	0.1845 (2)	0.4359 (4)	0.0456 (9)	
H3	0.284135	0.215146	0.470565	0.055*	
C4	0.1405 (2)	0.2382 (2)	0.3825 (4)	0.0362 (7)	
C5	0.1196 (2)	0.3491 (3)	0.3706 (4)	0.0398 (8)	
C6	0.1994 (2)	0.4266 (2)	0.4097 (4)	0.0387 (8)	
C7	0.2993 (2)	0.3988 (2)	0.3836 (5)	0.0485 (9)	
C8	0.1746 (2)	0.5218 (2)	0.4635 (4)	0.0401 (8)	
H8	0.107219	0.531495	0.469693	0.048*	
C9	0.2328 (2)	0.6115 (2)	0.5133 (4)	0.0392 (8)	
C10	0.1845 (2)	0.7056 (3)	0.5236 (4)	0.0453 (8)	
H10	0.116438	0.707623	0.505124	0.054*	
C11	0.2345 (2)	0.7946 (3)	0.5600 (4)	0.0487 (9)	
H11	0.200412	0.856348	0.564421	0.058*	
C12	0.3361 (2)	0.7934 (3)	0.5904 (4)	0.0439 (8)	
C13	0.3847 (2)	0.7009 (3)	0.5890 (4)	0.0497 (9)	
H13	0.452436	0.699000	0.613981	0.060*	
C14	0.3346 (2)	0.6118 (3)	0.5512 (4)	0.0482 (9)	
H14	0.368904	0.549987	0.550763	0.058*	
C15	0.4836 (3)	0.8892 (3)	0.6548 (6)	0.0774 (12)	
H15A	0.514909	0.862440	0.552324	0.116*	
H15B	0.501480	0.848084	0.759237	0.116*	
H15C	0.504330	0.958798	0.676470	0.116*	
N1	0.3776 (2)	0.3766 (2)	0.3524 (5)	0.0784 (11)	
O1	0.03542 (15)	0.37924 (17)	0.3351 (3)	0.0563 (6)	
O2	0.37932 (16)	0.88645 (17)	0.6199 (3)	0.0591 (7)	
S1	0.04432 (6)	0.15631 (7)	0.32754 (12)	0.0535 (3)	

### 3-(4-Methoxyphenyl)-2-[(thiophen-2-yl)carbonyl]prop-2-enenitrile Atomic displacement parameters (Å^2^)

**Table:**

	*U*^11^	*U*^22^	*U*^33^	*U*^12^	*U*^13^	*U*^23^
C1	0.068 (3)	0.039 (2)	0.057 (2)	−0.0065 (18)	0.0052 (19)	−0.0029 (17)
C2	0.050 (2)	0.046 (2)	0.064 (2)	0.0061 (17)	0.0009 (18)	0.0068 (18)
C3	0.040 (2)	0.036 (2)	0.060 (2)	0.0000 (15)	−0.0060 (16)	0.0078 (16)
C4	0.0377 (17)	0.0354 (19)	0.0354 (18)	0.0000 (15)	0.0020 (13)	−0.0007 (14)
C5	0.0283 (18)	0.052 (2)	0.0393 (19)	0.0012 (16)	0.0020 (14)	0.0007 (16)
C6	0.0305 (18)	0.040 (2)	0.0452 (19)	0.0078 (14)	−0.0014 (14)	0.0049 (15)
C7	0.035 (2)	0.036 (2)	0.074 (3)	0.0019 (16)	0.0008 (17)	−0.0039 (17)
C8	0.0286 (17)	0.044 (2)	0.047 (2)	0.0093 (15)	0.0011 (14)	0.0040 (16)
C9	0.0341 (18)	0.040 (2)	0.0431 (19)	0.0061 (15)	0.0011 (14)	0.0046 (15)
C10	0.0322 (18)	0.045 (2)	0.059 (2)	0.0077 (16)	0.0031 (15)	0.0004 (17)
C11	0.045 (2)	0.035 (2)	0.066 (2)	0.0147 (16)	0.0032 (17)	0.0017 (17)
C12	0.045 (2)	0.041 (2)	0.046 (2)	−0.0035 (17)	0.0040 (16)	−0.0012 (16)
C13	0.0329 (19)	0.050 (2)	0.066 (2)	0.0055 (17)	−0.0048 (16)	−0.0030 (18)
C14	0.038 (2)	0.040 (2)	0.065 (2)	0.0108 (16)	−0.0065 (16)	−0.0013 (17)
C15	0.054 (3)	0.073 (3)	0.105 (3)	−0.019 (2)	0.000 (2)	−0.005 (2)
N1	0.0395 (18)	0.055 (2)	0.141 (3)	0.0077 (16)	0.0098 (19)	−0.006 (2)
O1	0.0294 (13)	0.0551 (15)	0.0832 (17)	0.0061 (11)	−0.0054 (11)	0.0037 (13)
O2	0.0520 (16)	0.0452 (15)	0.0789 (18)	−0.0051 (12)	−0.0061 (13)	−0.0003 (12)
S1	0.0425 (5)	0.0562 (6)	0.0608 (6)	−0.0059 (4)	−0.0039 (4)	−0.0043 (5)

### 3-(4-Methoxyphenyl)-2-[(thiophen-2-yl)carbonyl]prop-2-enenitrile Geometric parameters (Å, º)

**Table:**

C1—C2	1.352 (4)	C9—C10	1.394 (4)
C1—S1	1.689 (4)	C9—C14	1.398 (4)
C1—H1	0.9300	C10—C11	1.361 (4)
C2—C3	1.405 (5)	C10—H10	0.9300
C2—H2	0.9300	C11—C12	1.389 (4)
C3—C4	1.375 (4)	C11—H11	0.9300
C3—H3	0.9300	C12—O2	1.359 (4)
C4—C5	1.473 (4)	C12—C13	1.374 (4)
C4—S1	1.718 (3)	C13—C14	1.367 (4)
C5—O1	1.226 (3)	C13—H13	0.9300
C5—C6	1.498 (4)	C14—H14	0.9300
C6—C8	1.351 (4)	C15—O2	1.428 (4)
C6—C7	1.433 (4)	C15—H15A	0.9600
C7—N1	1.142 (4)	C15—H15B	0.9600
C8—C9	1.447 (4)	C15—H15C	0.9600
C8—H8	0.9300		
			
C2—C1—S1	112.5 (3)	C14—C9—C8	125.1 (3)
C2—C1—H1	123.8	C11—C10—C9	121.8 (3)
S1—C1—H1	123.8	C11—C10—H10	119.1
C1—C2—C3	112.9 (3)	C9—C10—H10	119.1
C1—C2—H2	123.5	C10—C11—C12	120.2 (3)
C3—C2—H2	123.5	C10—C11—H11	119.9
C4—C3—C2	111.8 (3)	C12—C11—H11	119.9
C4—C3—H3	124.1	O2—C12—C13	125.3 (3)
C2—C3—H3	124.1	O2—C12—C11	115.7 (3)
C3—C4—C5	131.9 (3)	C13—C12—C11	119.0 (3)
C3—C4—S1	111.1 (2)	C14—C13—C12	120.7 (3)
C5—C4—S1	116.9 (2)	C14—C13—H13	119.7
O1—C5—C4	120.1 (3)	C12—C13—H13	119.7
O1—C5—C6	119.0 (3)	C13—C14—C9	121.3 (3)
C4—C5—C6	120.9 (3)	C13—C14—H14	119.3
C8—C6—C7	121.9 (3)	C9—C14—H14	119.3
C8—C6—C5	118.8 (3)	O2—C15—H15A	109.5
C7—C6—C5	119.2 (3)	O2—C15—H15B	109.5
N1—C7—C6	176.1 (4)	H15A—C15—H15B	109.5
C6—C8—C9	132.2 (3)	O2—C15—H15C	109.5
C6—C8—H8	113.9	H15A—C15—H15C	109.5
C9—C8—H8	113.9	H15B—C15—H15C	109.5
C10—C9—C14	116.9 (3)	C12—O2—C15	117.9 (3)
C10—C9—C8	118.0 (3)	C1—S1—C4	91.64 (16)
			
S1—C1—C2—C3	−0.3 (4)	C14—C9—C10—C11	3.2 (5)
C1—C2—C3—C4	0.3 (4)	C8—C9—C10—C11	−176.4 (3)
C2—C3—C4—C5	177.6 (3)	C9—C10—C11—C12	−0.8 (5)
C2—C3—C4—S1	−0.2 (3)	C10—C11—C12—O2	178.0 (3)
C3—C4—C5—O1	−172.8 (3)	C10—C11—C12—C13	−2.1 (5)
S1—C4—C5—O1	4.9 (4)	O2—C12—C13—C14	−177.5 (3)
C3—C4—C5—C6	4.4 (5)	C11—C12—C13—C14	2.5 (5)
S1—C4—C5—C6	−177.9 (2)	C12—C13—C14—C9	−0.1 (5)
O1—C5—C6—C8	21.7 (4)	C10—C9—C14—C13	−2.7 (5)
C4—C5—C6—C8	−155.6 (3)	C8—C9—C14—C13	176.8 (3)
O1—C5—C6—C7	−156.4 (3)	C13—C12—O2—C15	−0.9 (5)
C4—C5—C6—C7	26.4 (4)	C11—C12—O2—C15	179.0 (3)
C7—C6—C8—C9	−3.1 (5)	C2—C1—S1—C4	0.1 (3)
C5—C6—C8—C9	178.9 (3)	C3—C4—S1—C1	0.1 (2)
C6—C8—C9—C10	165.7 (3)	C5—C4—S1—C1	−178.1 (2)
C6—C8—C9—C14	−13.9 (5)		

### 3-(4-Methoxyphenyl)-2-[(thiophen-2-yl)carbonyl]prop-2-enenitrile Hydrogen-bond geometry (Å, º)

**Table:**

*D*—H···*A*	*D*—H	H···*A*	*D*···*A*	*D*—H···*A*
C8—H8···O1	0.93	2.40	2.779 (3)	104
C13—H13···N1^i^	0.93	2.51	3.394 (4)	158

Symmetry code: (i) −*x*+1, −*y*+1, −*z*+1.
